# Development but not diet alters microbial communities in the Neotropical arboreal trap jaw ant *Daceton armigerum*: an exploratory study

**DOI:** 10.1038/s41598-020-64393-7

**Published:** 2020-04-30

**Authors:** Manuela O. Ramalho, Christophe Duplais, Jérôme Orivel, Alain Dejean, Joshua C. Gibson, Andrew V. Suarez, Corrie S. Moreau

**Affiliations:** 1 Cornell University, Department of Entomology, Ithaca, NY 14850 USA; 2 CNRS, UMR Ecologie des Forêts de Guyane, AgroParisTech, CIRAD, INRA, Université des Antilles, Université de Guyane, Kourou, France; 3 Department of Entomology, University of Illinois at Urbana-Champaign, Urbana, Illinois USA; 4 Department of Evolution, Ecology and Behavior, University of Illinois at Urbana-Champaign, Urbana, Illinois USA; 5 Cornell University, Department of Ecology and Evolutionary Biology, Ithaca, NY 14850 USA

**Keywords:** Microbiology, Molecular biology

## Abstract

To better understand the evolutionary significance of symbiotic interactions in nature, microbiome studies can help to identify the ecological factors that may shape host-associated microbial communities. In this study we explored both 16S and 18S rRNA microbial communities of *D. armigerum* from both wild caught individuals collected in the Amazon and individuals kept in the laboratory and fed on controlled diets. We also investigated the role of colony, sample type, development and caste on structuring microbial communities. Our bacterial results (16S rRNA) reveal that (1) there are colony level differences between bacterial communities; (2) castes do not structure communities; (3) immature stages (brood) have different bacterial communities than adults; and 4) individuals kept in the laboratory with a restricted diet showed no differences in their bacterial communities from their wild caught nest mates, which could indicate the presence of a stable and persistent resident bacterial community in this host species. The same categories were also tested for microbial eukaryote communities (18S rRNA), and (5) developmental stage has an influence on the diversity recovered; (6) the diversity of taxa recovered has shown this can be an important tool to understand additional aspects of host biology and species interactions.

## Introduction

A prevailing question in studies of host-symbiont interactions is what are the host factors that affect their prokaryotic and eukaryotic microbial communities. Microbial communities are influenced by features including geography, host phylogeny, diet and stages of development^1–7^. However, no single factor consistently structures host-associated microbial communities across the tree of life. For example, in some ants diet explains substantial variation in gut microbial diversity^8–13^. In contrast, for *Pseudomyrmex* ants a specialized plant-based diet was not as important as their relative trophic position for explaining microbial abundance and diversity^14^. Understanding the patterns and exceptions affecting host-associated microbial diversity are critical to advancing our knowledge of these intimate relationships.

One of the most common benefits provided by symbiosis is nutrition^15^. Part of the evolutionary success of ants is attributed to food flexibility, which in some cases is directly linked to microbial symbionts^12,16,17^. Several ant species have well-established bacterial communities and some provide essential nutrients to the ants. For example, members of the Camponotini tribe (*Camponotus*, *Colobopsis* and *Polyrhachis*) rely on endosymbiotic *Blochmannia* to obtain nitrogen^3,4,18–20^, and species in the genus *Cephalotes* rely on intestinal symbionts to recycle nitrogen for the host^10,12,21^. However, the majority of ant taxa have not had their microbial communities studied, especially for eukaryotic microbes. In addition, understanding the factors that influence and modify the diversity of these associated microbes is important to understand their impact on the host.

*Daceton armigerum* is an arboreal trap-jaw ant with polymorphic workers that nests in the canopies of lowland Amazonian rainforests^22,23^. The success of arboreal ants is often attributed to their reliance on plant-based resources^12,24^, however this species preys on arthropods from the canopy to the ground^23^. In contrast to ants that rely on plant-based resources, gut endosymbionts of predatory species are often less diverse and lack nutrient-providing taxa^8,12,13^. Studies that examine how gut bacterial communities vary among life stages and castes, or with changes in diet, are also largely lacking from predatory ant species.

In this study, we examined bacterial (16S rRNA) and eukaryotic (18S rRNA) microbial communities of two colonies of *D. armigerum* from both wild caught individuals and individuals kept in the laboratory and fed on controlled diets. By investigating microbial communities from both wild and lab-reared individuals we investigated the following questions: (1) Are there differences in microbial communities between the colonies? (2) Do microbial communities vary with body size/castes that vary in behavior (foragers, nurses, etc.)? (3) Do immature stages (brood: larvae and pupae) host different microbial communities than adults?; and 4) Can microbial communities be changed by altering the diet? Data from 16S rRNA and 18Sr RNA from next-generation amplicon sequencing (NGS) were used to characterize the microbial communities and explore ecological questions related to the host.

## Materials and Methods

### Sample collection and determination of the different developmental stages

The 64 specimens in this study were from two colonies of *Daceton armigerum* (Latreille, 1802) collected in French Guiana in Cayenne (4.89847, −52.26999) (CSM3518) and in Kourou (5.17356, −52.65274) (CSM3520) (about 40 km apart). Voucher specimens were deposited in the scientific collections of the Field Museum of Natural History, Chicago, USA under voucher numbers FMNHINS3165241 (CSM3518) and FMNHINS3165242 (CSM3520). As these are arboreal ants that live in large living trees collecting colonies can be challenging. The colonies were found within branches and were carefully processed. Twenty-four specimens were collected in the field and immediately stored in 95% ethanol and kept at −20 °C until extraction of total DNA. To determine the caste/development stages we selected workers (with size variation to represent the polymorphism of adult workers (soldiers, medium and small)), males, pupae (with size variation - large, medium and small) and late stage larvae of different sizes. To test the influence of diet and if there is a difference in the bacterial community after being kept in the laboratory and fed a controlled diet, the remaining individuals were brought to the laboratory and fed a sugar water (20%) and cricket diet. These crickets were purchased through Armstrong Crickets (West Monroe, LA, USA) and immediately frozen before feeding them to the ants. After one month in the laboratory (“Time 1”), 11 live individuals were randomly selected, and their DNA was extracted. After another two months (“Time 2”), 29 additional individuals were again randomly selected and their DNA was extracted. We also examined the bacterial communities of the sugar water and crickets that constituted the diet of the live colonies when kept in the laboratory. All samples used for analysis were immediately collected into 95% ethanol in the field or lab and stored in 95% ethanol and kept at −20 °C until extraction of the total DNA (Table 1).

**Table 1:** Specimens used in the present study. In total, two colonies were sampled: CSM3518 and CSM3520.

**Collection code**	**Caste**	**Body part for extraction**	**Wild or laboratory specimens**	**qPCR**
CSM3518a	worker_large soldier	whole body	wild	107253.8121
CSM3518b	worker_medium size	whole body	wild	30850.60903
CSM3518c	worker_smaller size	whole body	wild	62768.27397
CSM3518d	worker_large soldier	gaster only	wild	116926.6404
CSM3518e	worker_large soldier	gaster only	wild	4182.870531
CSM3518f	worker_medium size	gaster only	wild	500148.3551
CSM3518g	worker_medium size	gaster only	wild	153368.7282
CSM3518h	worker_smaller size	gaster only	wild	62768.27937
CSM3518i	worker_smaller size	gaster only	wild	1134812.191
CSM3520a	worker_large soldier	whole body	wild	903603.3203
CSM3520b	worker_medium size	whole body	wild	14285.43608
CSM3520c	worker_smaller size	whole body	wild	77246.7959
CSM3520d	worker_large soldier	gaster only	wild	15707.7435
CSM3520e	worker_large soldier	gaster only	wild	7890.596614
CSM3520f	worker_medium size	gaster only	wild	16282.36467
CSM3520g	worker_medium size	gaster only	wild	14285.43608
CSM3520h	worker_smaller size	gaster only	wild	5.8822
CSM3520i	worker_smaller size	gaster only	wild	226.2195
CSM3520j	male	whole body	wild	277442.4457
CSM3520k	pupae	whole pupae	wild	105817.9332
CSM3520l	pupae	whole pupae	wild	22663.99421
CSM3520m	pupae	whole pupae	wild	305.6088804
CSM3520n	larvae	whole larvae	wild	143368.3363
CSM3520o	larvae	whole larvae	wild	277442.4457
CSM3518L1	worker_smaller size	gaster only	laboratory - time 2	7873.334527
CSM3518L2	worker_smaller size	gaster only	laboratory - time 2	54945.30271
CSM3518L3	worker_smaller size	gaster only	laboratory - time 2	13639.89950
CSM3518LE1	worker_smaller size	gaster only	laboratory - time 1	8288.498981
CSM3518LE2	worker_smaller size	gaster only	laboratory - time 1	75651.26549
CSM3518LL1	worker_smaller size	gaster only	laboratory - time 2	94425.62691
CSM3518LL2	worker_smaller size	gaster only	laboratory - time 2	169087.1036
CSM3518S1	worker_large soldier	gaster only	laboratory - time 2	74705.83026
CSM3518S2	worker_large soldier	gaster only	laboratory - time 2	759747.1508
CSM3518S3	worker_large soldier	gaster only	laboratory - time 2	221163.2453
CSM3518SE1	worker_large soldier	gaster only	laboratory - time 1	566448.8417
CSM3518SL1	worker_large soldier	gaster only	laboratory - time 2	486810.8988
CSM3518SL2	worker_large soldier	gaster only	laboratory - time 2	472345.5814
CSM3518W1	worker_medium size	gaster only	laboratory - time 2	24144.49178
CSM3518W2	worker_medium size	gaster only	laboratory - time 2	136091.6718
CSM3518W3	worker_medium size	gaster only	laboratory - time 2	255367.7115
CSM3518WE1	worker_medium size	gaster only	laboratory - time 1	54234.94541
CSM3518WE2	worker_medium size	gaster only	laboratory - time 1	236922.6482
CSM3518WL1	worker_medium size	gaster only	laboratory - time 2	238852.7744
CSM3518WL2	worker_medium size	gaster only	laboratory - time 2	301598.7693
CSM3520L1	worker_smaller size	gaster only	laboratory - time 2	11303.30712
CSM3520L2	worker_smaller size	gaster only	laboratory - time 2	28837.72747
CSM3520L3	worker_smaller size	gaster only	laboratory - time 2	92702.01409
CSM3520LE1	worker_smaller size	gaster only	laboratory - time 1	947.0076534
CSM3520LE2	worker_smaller size	gaster only	laboratory - time 1	113503.6583
CSM3520LL1	worker_smaller size	gaster only	laboratory - time 2	115.6949113
CSM3520LL2	worker_smaller size	gaster only	laboratory - time 2	105853.8457
CSM3520S1	worker_large soldier	gaster only	laboratory - time 2	96673.77109
CSM3520S2	worker_large soldier	gaster only	laboratory - time 2	52052.75086
CSM3520S3	worker_large soldier	gaster only	laboratory - time 2	30149.71022
CSM3520SE1	worker_large soldier	gaster only	laboratory - time 1	332361.84409
CSM3520SE2	worker_large soldier	gaster only	laboratory - time 1	345596.6589
CSM3520SL1	worker_large soldier	gaster only	laboratory - time 2	53984.08424
CSM3520SL2	worker_large soldier	gaster only	laboratory - time 2	1303908.326
CSM3520W1	worker_medium size	gaster only	laboratory - time 2	390420.8943
CSM3520W2	worker_medium size	gaster only	laboratory - time 2	27682.57562
CSM3520W3	worker_medium size	gaster only	laboratory - time 2	65294.58984
CSM3520WE1	worker_medium size	gaster only	laboratory - time 1	2213.643473
CSM3520WE2	worker_medium size	gaster only	laboratory - time 1	1208744.225
CSM3520WL1	worker_medium size	gaster only	laboratory - time 2	418266.1593
cricket1	diet1	diet1	diet1	129254.9951
cricket2	diet1	diet1	diet1	13949361.99
cricket3	diet1	diet1	diet1	50693.36052
sugarwater1	diet2	diet2	diet2	60.51831745
sugarwater2	diet2	diet2	diet2	40.853
sugarwater3	diet2	diet2	diet2	53.125

### DNA Extraction and Bacterial DNA Sequencing

Workers had DNA extracted in two ways: whole body and some specimens containing only the gasters (abdomens). Also, total DNA was extracted from immature samples (whole immature) when available (see Table 1) using the DNeasy PowerSoil Kit (Qiagen, USA) with the modification of a beat-beating step and addition of Proteinase K following the protocol of Rubin *et al*.^25^. Filtered pipette tips and sterile techniques were applied to avoid contamination following Moreau^26^. Four blank samples were also included as negative controls. Amplification of the V4 region of 16S rRNA (515 F/806 R primers) and V1–V2 region of 18S rRNA from microbial eukaryotes (F04/R22 primers) were performed as described in Caporaso *et al*.^27^ and Creer *et al*.^28^, respectively, following the Earth Microbiome Project (EMP) protocol (http://www.earthmicrobiome.org/protocols-and-standards/). Three PCR reactions were performed per sample (triplicate), each 25 μl PCR reaction contained 12 μl of PCR water (Certified DNA-free), 10 μl of 5 Prime HotMasterMix (1×) (5 PRIME, Gaithersburg, USA), 1 μl of forward primer (5 mM concentration, 200 final pM), 1 μl Golay barcode tagged reverse primer (5 mM concentration, 200 pM final) and 1 μL of template DNA (>0.20 ng/ μl), under the following conditions 94 °C for 3 min, with 35 cycles at 94 °C for 45 s, 50 °C for 60 s, and 72 °C for 90 s, with a final cycle of 10 min at 72 °C. Confirmation of the efficiency of the amplification was performed by agarose gel electrophoresis (1%). The samples were quantified via qPCR and Qubit (Thermo Fisher Scientific) with the High Sensitivity Assay Kit (Life Technologies Corp., Carlsbad, USA), and only then were all samples pooled. Each pool containing 100 μL was cleaned using the QIAquick PCR Purification Kit (Qiagen, USA), following the manufacturer’s recommendations. The molarity of the pool was determined and diluted down to 4 nM, denatured, and then diluted to a final concentration of to 6.75pM with a 10% PhiX for sequencing at Argonne National Laboratory (Lemont, Illinois, USA). Two separate runs (16S and 18S rRNA) were performed with the MiSeq Illumina V3 Reagent Kit 600 Cycles (300 × 300) using the custom sequencing primers and procedures described in the supplementary methods in Caporaso *et al*.^27^ for 16S rRNA and Creer *et al*.^28^ for 18S rRNA. All raw sequence data are publicly available NCBI SRA accession number PRJNA559936 and BioSample SUB5901646.

### Bacterial Quantification

qPCR was performed on real-time CFX Connect equipment (Bio-Rad, Hercules, USA) using the SYBRAdvanced 2X (Bio-Rad) SYBR green supermix and 2 μL of DNA to verify the total amount of bacteria present in each sample. For this, the 16S rRNA gene was amplified using the universal primers 515 f (5′-GTGCCAGCMGCCGCGGTAA) and 806r (5′-GGACTACHVGGGTWTCTAAT) (http://earthmicrobiome.org/emp-standard-protocols/16s/). Standard curves were generated from serial dilutions of linearized plasmids containing *E. coli* 16S rRNA inserts, following the same parameters of Rubin *et al*.^25^. All qPCRs were satisfactory and had R2 from 90% and 110%. All samples were analyzed in triplicates, including blank samples (Table 1).

### Bioinformatic analysis

Demultiplexed sequence data were analyzed using the Qiime2-2019.1^29^ with plugin demux (https://github.com/qiime2/q2-demux). Sequence quality control and feature table construction were performed through the dada2 plugin^30,31^. Taxonomic assignment was conducted with the SILVA_132_QIIME database and the ASV (amplicon sequence variants) were selected with 99% identity^32,33^ and to generate the taxonomy table paired-end sequence reads were trimmed in the v4 region of 16S rDNA with the 515F/806R primers. Thereby, our own classifier was created using the “feature-classifier fit-classifier-naive-bayes” command. Once the classifier was obtained, the reads (rep-seqs) were classified by taxon using the “feature-classifier classify-sklearn” command^34^.

After obtaining the feature table, the filtration of negative controls was performed with the Decontam package^35^ using R software^36^. Following the recommendations of Łukasik *et al*.^11^, from the extraction to the sequencing of the library, four negative controls were included. These controls served to remove contaminants from the *D. armigerum* samples. In the Decontam package^35^ the prevalence method was the one that managed to better filter our data and removed the largest number of contaminants and therefore was applied to our samples. These decontaminated data were then brought back into Qiime2 where subsequent filtering also removed mitochondria, chloroplast and Hymenoptera sequences from the feature-tables. The feature-table summarize command creates a visual summary of the 16S rRNA and 18S rRNA data. For each dataset the alignment was performed using the align-to-tree-mafft-fasttree command^37^. The microbial phylogeny was reconstructed^38^ and alpha and beta diversity analyses were performed by using the “qiime feature-table core-features” command. These results were visualized with the software emperor^39^. We used the cutoff of 4,000 and 1,000 sequences for 16S rRNA and 18S rRNA respectively in downstream analyses, because normalization is necessary for valid comparisons of abundance and diversity.

Statistical analyses were conducted with the Permanova Test with Unifrac distance which is a phylogenetic metric. The “diversity beta-group-significance” command^40^ tested whether the distances between samples within a given group, for example if the samples from Colony 1 are more similar to each other than the samples from Colony 2. In all tests the parameter – “p- pairwise” was applied to determine which specific pairs of groups differ from each other, for example, within the Caste category, all options were tested in pairs. To visualize the relationships between microbes associated with *D. armigerum*, we implemented an analysis of multidimensional nonmetric scaling (NMDS) and related statistics in the PAST3 software package^41–43^. In addition, through Simper’s analysis we explored the contribution of the main ASVs in the present study^41^.

To visualize networks of bacterial communities among the workers and brood sampled in the present study phyloseq^44^ and ggplot2^45^ in R software^36^ were used to show connections between samples. A heatmap was constructed to visualize the main two groups of bacteria, Rhizobiales and Entomoplasmatales and their strains, with the ‘qiime feature-table heatmap’ plugin^46^. Phylogenetic reconstruction of the major ASVs (amplicon sequence variants) from our study belonging to the Rhizobiales and Entomoplasmatales were performed. For this analysis we also included bacteria with close Blast hits from GenBank. We also took into account the hosts of these bacteria from GenBank. All sequences were edited in the Bioedit Sequence Alignment Editor^47^ and aligned with the ClustalW tool^48^. We implemented a maximum likelihood analysis using PhyML 3.0^49^ on the CIPRES web portal^50^. The GTR + G model of molecular evolution was used to infer the two phylogenies. Branch lengths and bootstrap support are reported. To facilitate visualization the ASVs found in the present study are highlighted in red and the phylogenies have been edited in FigTree v1.3.1 (http://tree.bio.ed.ac.uk/software/figtree/).

## Results

### rRNA

**Figure 1:**
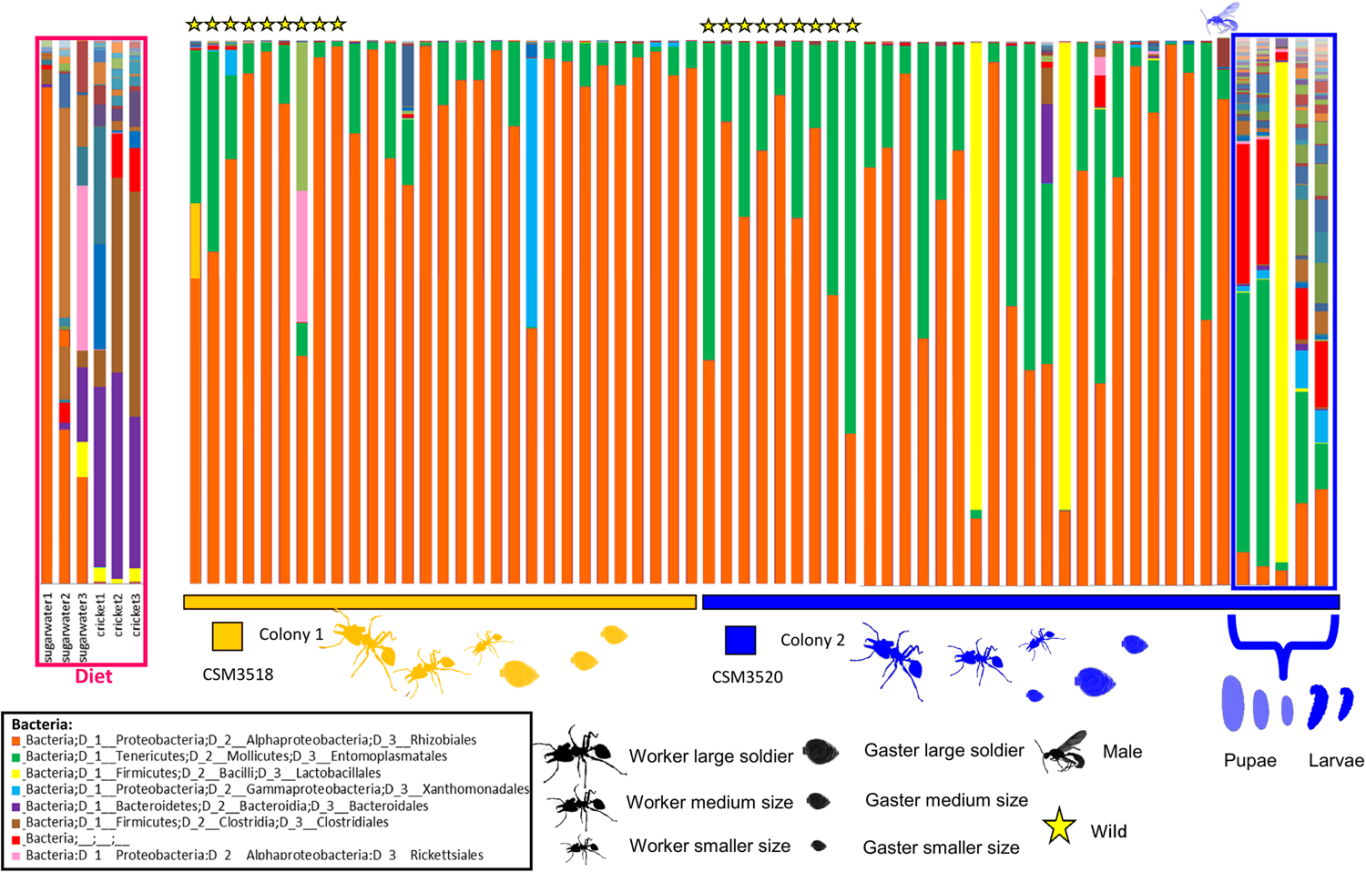
Alpha diversity of the main bacterial ASVs found in two colonies of *Daceton armigerum* with 16S rRNA amplicon sequencing. The samples were grouped according to the colony from which they belong. Bar graphs for each library (one column = community from a single sample) show the percentage of sequence reads classified to selected 99% similarity. Each color represents a distinct bacterium. Yellow stars differentiate wild caught samples.

The depth of the samples was rarefied to 4,000 reads and sequencing reached the plateau (Supplemental Information1). We obtained 1,837 ASVs from 2,581,996 reads from the 70 samples (64 from *D. armigerum*, and 6 from diets) ranging from 949 to 93,032 reads. To facilitate the interpretation and visualization of the bar-plot graph, the hierarchical taxonomic level chosen was Order. The main bacteria found were Rhizobiales (69%), followed by Entomoplasmatales (17%), Lactobacillales (4%) and others in smaller amounts (Fig. 1). There were differences in the relative contribution of these groups between colonies, and between brood and workers, with immatures having a low relative abundance of Rhizobiales compared to workers.

**Figure 2:**
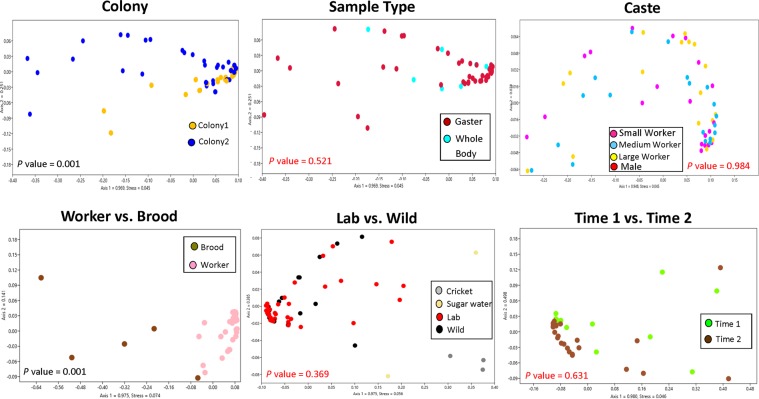
Beta diversity data with NMDS plots of bacterial communities of *Daceton armigerum* samples.

We found high alpha diversity in the *Daceton* samples, with the larvae having the greatest diversity as assessed by the Shannon Index (Supplemental Information 2). Beta diversity analysis was performed using UniFrac weighted distance matrices to take into account the abundance of bacteria, and NMDS were used to graphically represent the different clusters of the *Daceton armigerum* samples (Fig. 2). Although difficult to visualize, bacterial communities of workers varied between Colony 1 and Colony 2 (P-value = 0.001, pseudo-F = 8.163). To further explore the differences between colonies, additional analyses were conducted separately for samples from the Wild, Time 1 and Time 2. With the exception of Time 1, the difference between the colonies remained (Wild: P-value = 0.01, pseudo-F = 4.949; Time 1: P-value = 0.06, pseudo-F = 1.727; Time 2: P-value = 0.002, pseudo-F = 4.755). In addition, through the Simper analysis, we found that the main bacteria responsible for the difference found in each group (Wild, Time 1 and Time 2) is Lactobacillales, which have an increased abundance in the samples kept in the laboratory (Supplemental Information 3).

**Figure 3:**
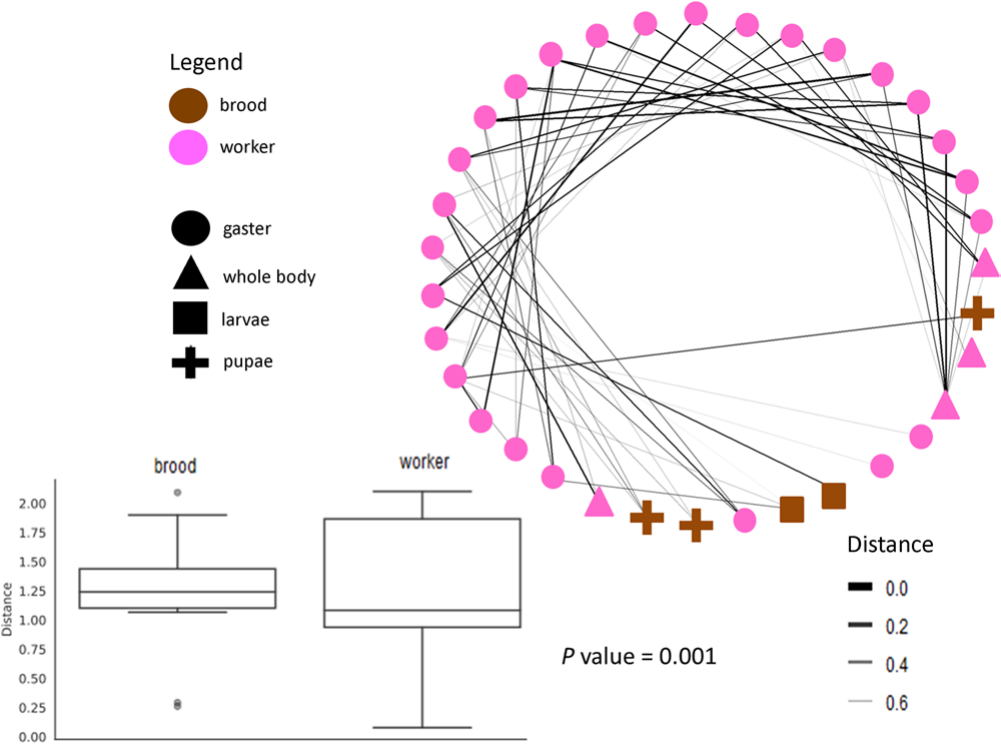
Network analysis of *Daceton armigerum* bacteria from workers (pink) and brood (brown) are identified by the sample type. The graph below shows the difference (P-value = 0.001) between workers and brood.

Colony 2 (CSM3520) included immature samples (brood), and we found differences between the workers and immatures (P-value = 0.001, pseudo-F = 42.283) (Figs. 1–3). However, there was no difference among workers of different sizes (P-value = 0.984, pseudo-F = 0.297), the sample type (gaster or whole body) (P-value = 0.521, pseudo-F = 0.801), “Lab vs. Wild” (P-value = 0.369, pseudo-F = 1.080) and “Time 1 vs. Time 2” (P-value = 0.631, pseudo-F = 0.661). Although few whole bodies were included in this study compared to gaster samples, these results suggest that the tissue analyzed is not an important factor to consider when investigating bacterial communities as they are likely dominated by bacteria in the digestive tract. In addition, the most surprising result of this study was that no difference was found between the wild caught and laboratory fed samples, even across two time points: Time 1 (early) and Time 2 (late). This could be an indication that diet does not influence the bacterial community found in *D. armigerum* (Fig. 2).

The bacterial quantification results from the qPCR of 16S rRNA (bacterial) shows there is no difference for any of the categories tested in the present study: Colony (ANOVA, P-value = 0.650), Sample Type (P-value = 0.830), Caste (P-value = 0.270), Developmental Stage (workers and immature) (P-value = 0.570), Lab vs. Wild (P-value = 0.480) and Time 1 vs. Time 2 (P-value = 0.570)(Supplemental Information 4).

To illustrate the complexity of the symbiotic interactions from the significant differences found between workers (colored in pink) and brood (colored in brown), a network analysis was performed, where each node represents a host sample identified by the sample type and the connections illustrate shared bacterial communities as distances (Bray-Curtis). Note that the workers are different from the immatures (Fig. 3).

**Figure 4:**
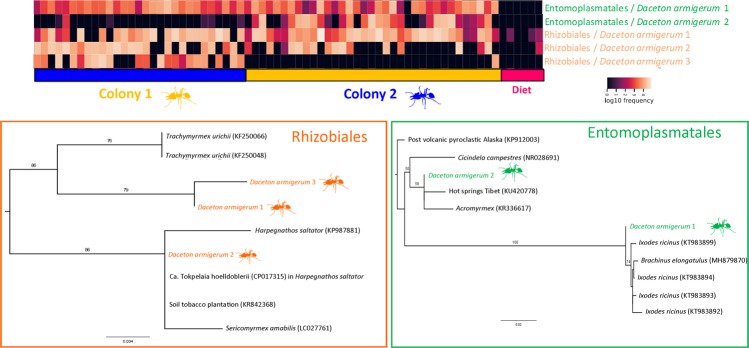
Main bacterial strains found in *Daceton*: heatmap and phylogenetic tree of the main ASVs with their closest sequences available in GenBank. The colors in the heatmap indicate variation in the relative abundance of different bacteria and strains in *Daceton armigerum*. In the heat map, each column represents a sample. The samples were grouped according to the colonies that they belong. Maximum likelihood phylogenies of the 16S rRNA region of the main bacterial symbionts of this study along with the closest matches in GenBank. Bootstrap support is shown on branches. The labels are given with host, where they were found, GenBank accession number (GenBank sequences) and collection code (sequences generated in the present study are colored in orange for Rhizobiales and green for Entomoplasmatales).

For the heatmap analysis we focused on the main bacteria found in *Daceton armigerum*: Rhizobiales and Entomoplasmatales and their strains (Fig. 4, Supplemental Information 5). The lab diets had a very distinct community from the *D. armigerum* samples (P-value = 0.001, pseudo-F = 14.164). These bacteria occur in high abundance in the two colonies, with some exceptions: Entomoplasmatales/*Daceton armigerum* 2 is much more common in Colony 2. Besides that, there was not a clear distribution pattern of the other bacteria, that is, these main bacteria occur in different Colonies, Sample Type, Caste, Workers vs. Brood, Lab vs. Wild and Time 1 vs. Time 2 of *D. armigerum*. Therefore, we examined the most common ASVs from the two most abundant groups (Rhizobiales and Entomoplasmatales) and Blasted them against GenBank to infer phylogenies for each of these groups to see if the *Daceton* microbes are part of an ant specific clade or independent acquisitions of these groups. As we can see for the two reconstructed phylogenetic trees, for both the different strains of Rhizobiales and for the strains of Entomoplasmatales, these did not group into *Daceton* specific clades. This suggests that these bacteria were acquired independently during various time points. In the phylogeny of Entomoplasmatales, the strains found in the present study were not clustered in a specific clade of ants or even insects. Although the Rhizobiales bacteria found in *Daceton* do not cluster in a single clade, all the similar bacteria found in Genbank belong to Formicidae (ants), except for a sample of soil from a tobacco plantation (KR842368).

### 18S rRNA

**Figure 5:**
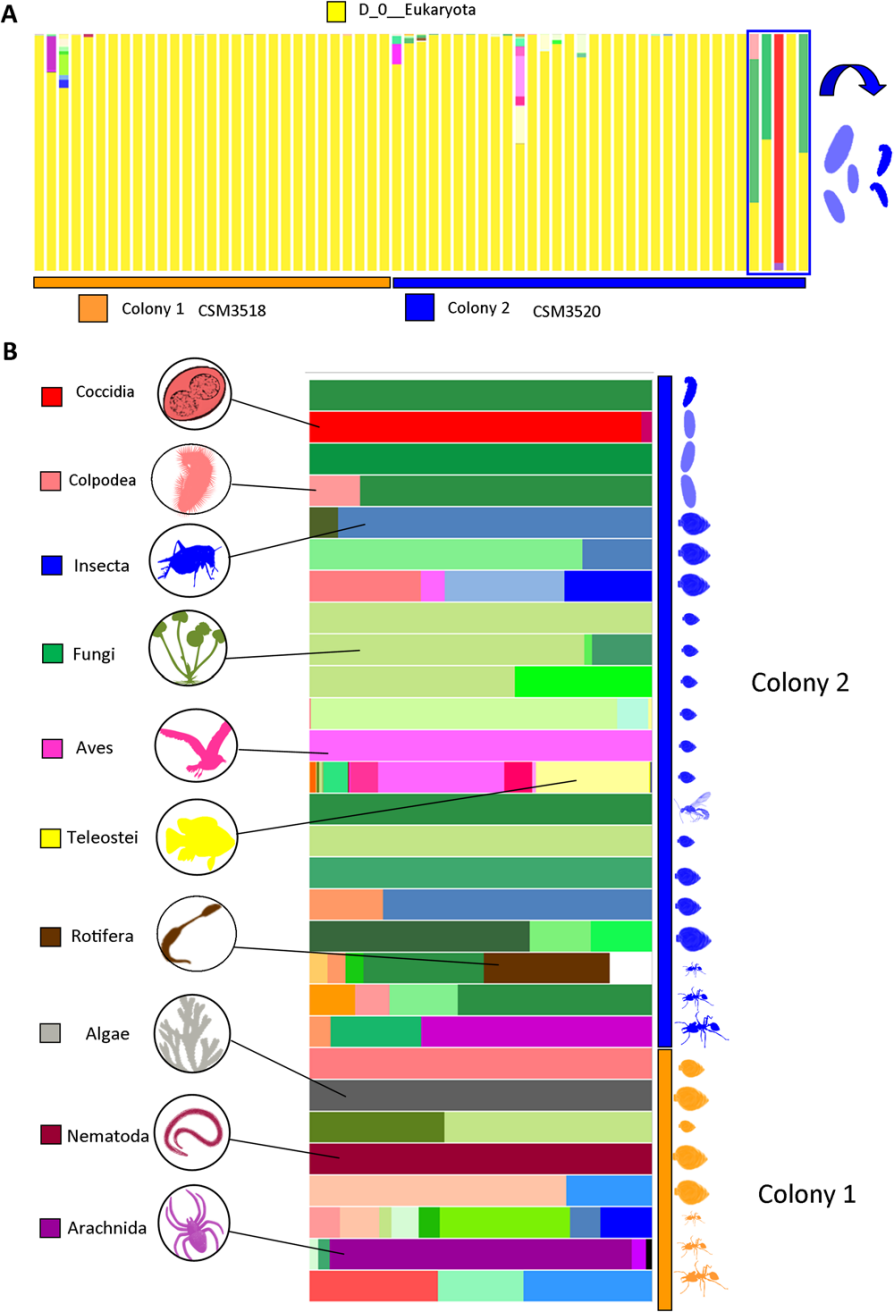
Alpha diversity of non-Hymenoptera microbial eukaryotes associated with *Daceton armigerum* clustered at 99% similarity in QIIME2. Each bar represents the microbiome profile associated with a single ant and each color represents a distinct non-hymenopteran microbial eukaryote. (**A**) Graph shows relative abundance of non-Hymenoptera 18S rRNA sequences. Note that the main taxon could only be identified to Eukaryota in the available database and that there is a clear separation of the immatures compared to workers. (**B)** Samples only resolved to “Eukaryota” were removed to facilitate the visualization of the other microbial eukaryotes identified.

Our rarefaction curves, at 1,000 read depth after excluding all hymenopteran data, show our sequencing coverage of the microbial eukaryotes communities appears adequate for most samples (Supplemental Information 6) after 24 samples were excluded since they did not contain the minimum number of reads as used for rarefaction of the remaining samples (see Supplemental Information 7). From the remaining samples 327 ASVs were recovered from 191,455 reads (some samples failed to amplify and could not be sequenced) ranging from 1,022 to 30,393 reads. The bar-plot graph with hierarchical level of species shows relative abundance of microbial eukaryotes and that the most abundant ant-associated eukaryote group was “Eukaryota” (meaning these sequences could not be distinguished below the highest hierarchical level) followed by *Adelina* (Coccidia), aves, teleosts and others in lower abundances. However, it is clear that the immatures possess a greater alpha diversity when compared with the workers (Fig. 5A). To facilitate visualization the samples that could only be identified to “Eukaryota” were excluded (Fig. 5B). The results of beta diversity (UniFrac weighted distance matrices) revealed that besides “Workers vs. Brood” (P-value = 0.043, pseudo-F = 23.066), the microbial eukaryotes associated with *D. armigerum* were not significantly grouped by any category: Colony (P-value = 0.127, pseudo-F = 1.536), Sample Type (P-value = 0.858, pseudo-F = 0.093), Caste (P-value = 0.320, pseudo-F = 0.756), Lab vs. Wild (P-value = 0.600, pseudo-F = 0.785) and Time 1 vs. Time 2 (P-value = 0.827, pseudo-F = 0.385). However, interestingly microbial eukaryotes appear associated with *D. armigerum*, such as fungi (each one colored with a different shade of green), insects (each one colored with a different shade of blue) and even teleosts (each one colored with a different shade of yellow). A taxon that also caught our attention was an entomopathogenic nematode *Heterorhabditis zealandica* (colored in dark red). The complete microbial eukaryotes list can be found in Supplemental Information 8.

## Discussion

Studies of 16S rRNA and 18S rRNA microbial communities have helped scientists understand the ecological and evolutionary factors that structure host-associated microbial communities^7,51^. Especially in ants, many studies have found diverse host-associated microbes and discovered aspects of host biology that impact microbial communities^3,4,6,10,12,14,21,52–54^. However, most of these studies focus on bacterial 16S rRNA. Therefore, our findings are innovative and explored connections among 16S rRNA and 18S rRNA microbial communities and developmental stage, colony interaction, sample type, and diet of *Daceton armigerum* host. It is incredibly rare to find and collect colonies of this species, which demonstrate how valuable our data are. However, we recognize that our study was conducted with two colonies and is therefore an exploratory study.

Previous studies have shown associations of Rhizobiales and Entomoplasmatales bacteria in ants^10–12,54^ including in *Daceton armigerum*^13^. Across the ants Rhizobiales was acquired at least five times independently and this may have facilitated the evolutionary success of several taxa by permitting them to occupy new niches^12^. Many studies have documented the association of this bacterium in hosts with herbivorous habits as is the case in *Dolichoderus* and *Cephalotes*^10,12,55^. For *Cephalotes* this bacterium facilitates the recycling of urea and obtaining nitrogen for the host^10,12^. However, a herbivorous diet is apparently not required for association in ants, as Rhizobiales has been found in carnivorous and omnivorous ant hosts, such as *Hapergnathos saltator, Pheidole, Paraponera clavata* and some army ants^11,12,56,57^. Although the role of this bacterium is not fully understood in ants with a high protein diet, a possible benefit could be the ability to degrade proteins^58^. This is supported by evidence from the genome of *Candidatus Tokpelaia holldoblerii* obtained from *Hapergnathos saltator* since genes involved in protein degradation, urea hydrolysis and vitamin biosynthesis were found^57^.

The Rhizobiales sequences found in *Daceton* had a high similarity to the bacterial genome of *Ca. Tokpelaia holldoblerii* found in the ant *Hapergnathos saltator*. In addition, in a study of bacterial communities of *Pseudomyrmex* ant Rubin *et al*.^14^ also found a symbiotic relationship with Rhizobiales and were able to analyze the genes present and found the complete urea recycling pathway suggesting a function of this bacterium for *Pseudomyrmex*. Unfortunately with our 16S rRNA amplicon data we cannot access the functions of these bacteria and the different strains may have different functions^11^ so understanding the function of these associations in nature with different ant taxa is still far from being complete.

Entomoplasmatales was also commonly found in this study and our results show that there is no specificity of this bacterium either with the host *D. armigerum* or with other insects, since similar strains recovered from Genbank also showed similarity with bacteria found in hot springs and post volcanic pyroclastic. This bacterium has also been found in several other ant taxa such as *Cephalotes varians*^59^, army ants^9,11^ and *Atta texana*^60^. It was also found in abundance in the post pharyngeal gland of *Atta sexdens*^54^ and in *Pseudomyrmex gracilis* generalist larvae^14^. More studies are needed to understand the importance of this bacterium in ants.

Lactobacilialles was one of the most common bacteria found in the present study. Although Lactobacilialles was found in both wild caught and lab reared samples it was in low abundance in wild caught samples and became very abundant in lab reared samples. These bacteria have already been associated with several other ant studies^4,54,61,62^, but its function in this group is not yet fully understood. However, studies have demonstrated an effect of this bacteria on the immune system of insects^63^, suggesting that in environmental stress conditions (e.g. pesticides or kept in the laboratory), it may increase the relative abundance of *Lactobacillus*^64,65^. This scenario could be a possible explanation for the increase of Lactobacilialles associated with *D. armiregum* kept in the lab.

As shown in this present study, for the two included colonies of *Daceton* we found some signature of colony specific bacterial communities, as well as distinct microbial communities between stages of development as has been seen in *Camponotus*^3^. Similar results across colonies were also recovered from bumble bees^66,67^. This is likely because all of these insects are eusocial and live in densely populated colonies with highly related individuals^68^ and this can act as a facilitator for the homogenization of the microbiota in the colony. Although we recognize that in the present study only two colonies were analyzed, we did find colony level differences. *D. armigerum* colonies are rare to collect, especially containing immatures, which makes the present study even more valuable. In addition, our data are also consistent with Rubin *et al*.^25^ who showed that the bacterial communities of larvae have high alpha diversity compared to that of workers. This pattern is most likely because at this stage of development sibling workers feed them intensively from food outside the nest before the pupae stage, being an excellent pathway for acquisition of transient and environmental microbial communities. In addition, larvae are mostly fed with prey items collected by foraging workers whereas adults feed only on liquids such as hemolymph from prey, extrafloral nectar and honeydew from hemipterans.

Several studies of mammals have shown the influence of diet in structuring the host-associated bacterial community^2,51^. Diet also seems to influence some groups of ants^8–13^. However, Rubin *et al*.^14^ found that for *Pseudomyrmex* trophic level has a stronger impact on the bacterial community than a specialized diet. In this study, the authors also argue that the bacterial communities associated with *Pseudomyrmex* showed more variability than in *Camponotus* and *Cephalotes*. In this study, although ants were exposed to several exogenous microbes with modified diet, they were not able to settle (colonize) in the *D. armigeum* host. In addition, there was no significant difference between the individuals caught in the wild or fed on a controlled diet across two time periods. Several factors can influence the success of colonization by exogenous microbes and the presence of a native microbiota offering resistance is one of the main lines of defense, especially in the case of hosts with high diversity communities^69^, as may be the case in this study. In addition, we acknowledge that an investigation of the effects of the individuals caught in the wild or fed on a controlled diet requires a greater understanding of the composition of the wild diet of the two colonies that were sampled here, but there is little data on the general biology of this omnivorous species, which further increases the importance of the 18S rRNA data from the present study. Also, confounding effects are numerous with not only the diet but temperature, humidity, density being different between the field and lab conditions and these changes may have influenced the composition of the microbiome. However, our data is consistent and we did not detect any difference between the lab-adapted and wild individuals.

Several studies with mice have already demonstrated the importance of genetic background shaping the bacterial community^70–72^. Specifically for ants, Hu *et al*.^21^ attributes the variation of bacterial communities among *Cephalotes varians* colonies to genetic variability of the host. Our data suggest that the same may be occurring in the bacterial community of *D. armigerum*. Therefore, in general, there are a colony-level signatures and this may occur due to the genetic background of the host. In addition, these differences between colonies are so stable that they remain even after dietary changes. Although only two colonies from different locations were sampled, our results show that there are significant differences between them. However, we recognize that many factors may be driving the differences among colonies (i.e., genetic differences or site differences).

There are few studies on any aspect of *Daceton* biology^22,23,73^ and there is still much to discover about this species and its interactions. To understand more about the microbial interactions in this host species we surveyed the bacterial and microbial eukaryotic communities. Data from eukaryotic microbial communities can be very enlightening regarding the ecology, biology and behavior of the host^7^. For *Daceton* some of the categories investigated (Colony, Sample Type, Caste, Lab vs. Wild and Time 1 vs. Time 2) do not influence the pattern retrieved from the 18S rRNA data set, except when compared between immatures and workers, a result we also see reflected in the bacterial community data. Our analysis of the 18S rRNA resulted in several unexpected taxa associated with *D. armigerum*. However, as this species is omnivorous on living and dead material, we cannot exclude the possibility that it actually fed on some of these taxa.

Interestingly in the immature samples we recovered Botryosphaeriales fungi, *Adelina* (Coccidia) and Colpodea ciliates. Botryosphaeriales fungi are endophytic and can occur worldwide^74^ and as *Daceton* nests exclusively in trees they may acquire these microbes as they excavate their nest cavities. Colpodea (Bromeliothrix) is currently only known from bromeliad tanks^75^ and the mechanism these encysted species are dispersed is not fully understood^76^, but our findings suggest that *D. armigerum* could be assisting in the dispersion of these cysts if these ciliates are common in tropical bromeliads too. *Adelina* is a genus of Coccidians ciliates and are exclusively insect parasites and although they have not been described in Hymenoptera our results suggest they may infect *Daceton*, although we cannot rule out that these were ingested with infected prey items. Another important highlight of our 18S rRNA data set is the presence of the obligate entomopathogenic nematode *Heterorhabditis zealandica*, as nematodes are often used for insect control^77,78^. Although intriguing, this nematode was found only in high relative abundance in one gaster of a worker, so we cannot argue about the impact on the health of the colony. These 18S rRNA findings are unique and demonstrate the potential of this method to capture microbial eukaryotic communities in hosts, but whether these relationships are a symbiosis, parasitism or even just larval food, further studies are needed to explore these observations^7^.

## Conclusion

This is the first ant study to address these two host-associated microbial libraries together (16S rRNA and 18S rRNA) and our data highlight that studies like these have the ability to reveal important information regarding the diversity of microbial communities that can infect hosts and which host factors may influence microbial associations. Although we did not find differences between bacterial quantification (qPCR of 16S rRNA), we were able to verify that colony and developmental stages are factors that contribute to and explain the diversity of the bacterial community found in *D. armigerum*. However, sample type (gaster or whole body) and individuals kept in the laboratory with a restricted diet in comparison with wild caught siblings showed no differences in their bacterial communities. All these categories were also tested for microbial eukaryote communities, but apart from differences between the immature and the workers these factors seem to have no influence on the diversity recovered. However, the diversity of taxa recovered through 18S rRNA has shown to be an important tool to understand aspects of the behavior, health and ecology of the host.

## Supplementary information

Supplementary Information 1.

Supplementary Information 2.

Supplementary Information 3.

Supplementary Information 4.

Supplementary Information 5.

Supplementary Information 6.

Supplementary Information 7.

Supplementary Information 8.

## Data Availability

All raw sequence data are publicly available NCBI SRA accession number PRJNA559936 and BioSample SUB5901646.
