# Supplementary figures and images for: Development but not diet alters microbial communities in the Neotropical arboreal trap jaw ant Daceton armigerum: an exploratory study
Source: Sci Rep. 2020 Apr 30;10:7350. doi: 10.1038/s41598-020-64393-7 (PMC7192945; doi:10.1038/s41598-020-64393-7)

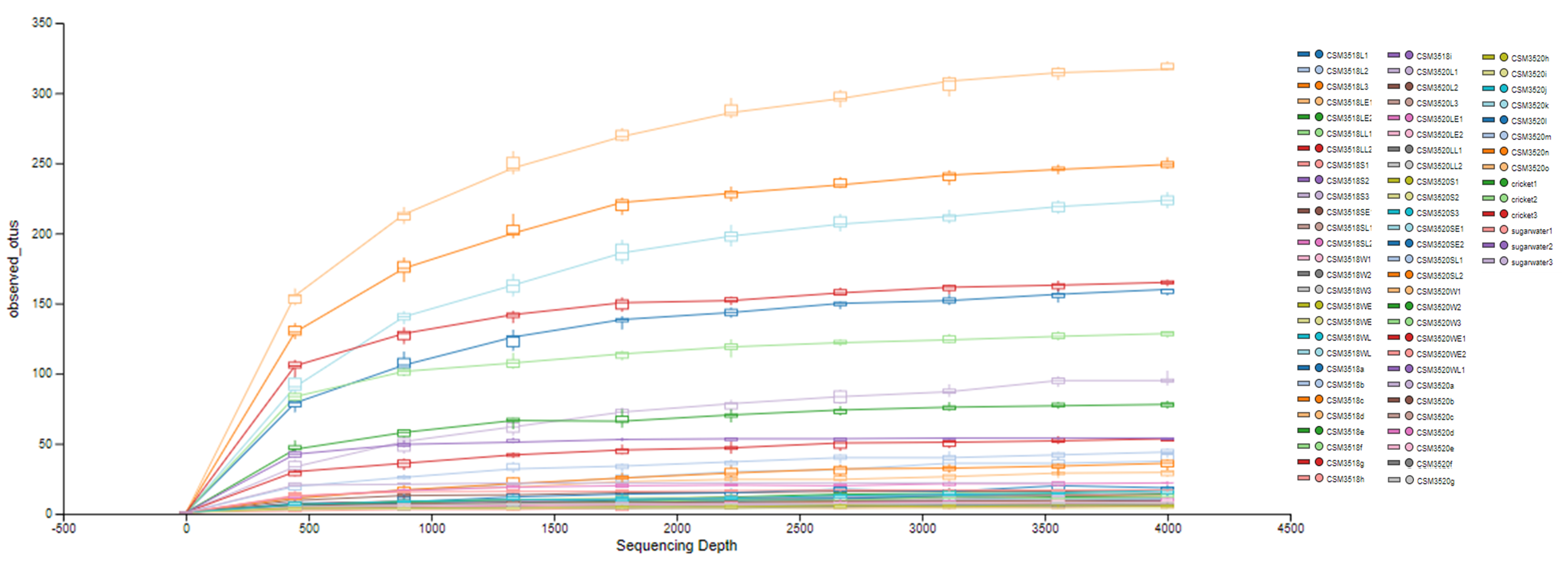

Supplement: Supplementary file 1 — Supplementary Information 1. [file 41598_2020_64393_MOESM1_ESM.tif]

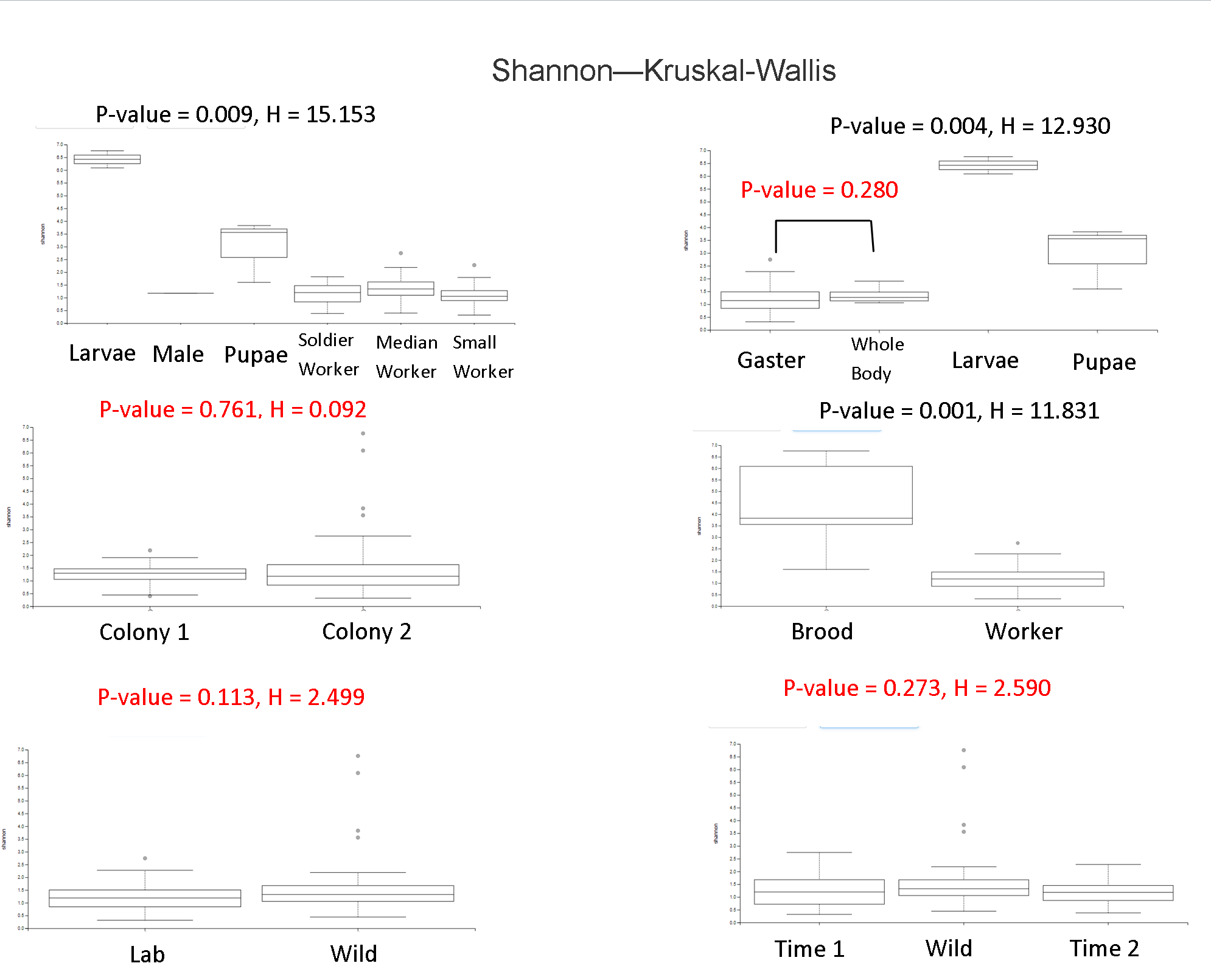

Supplement: Supplementary file 2 — Supplementary Information 2. [file 41598_2020_64393_MOESM2_ESM.tif]

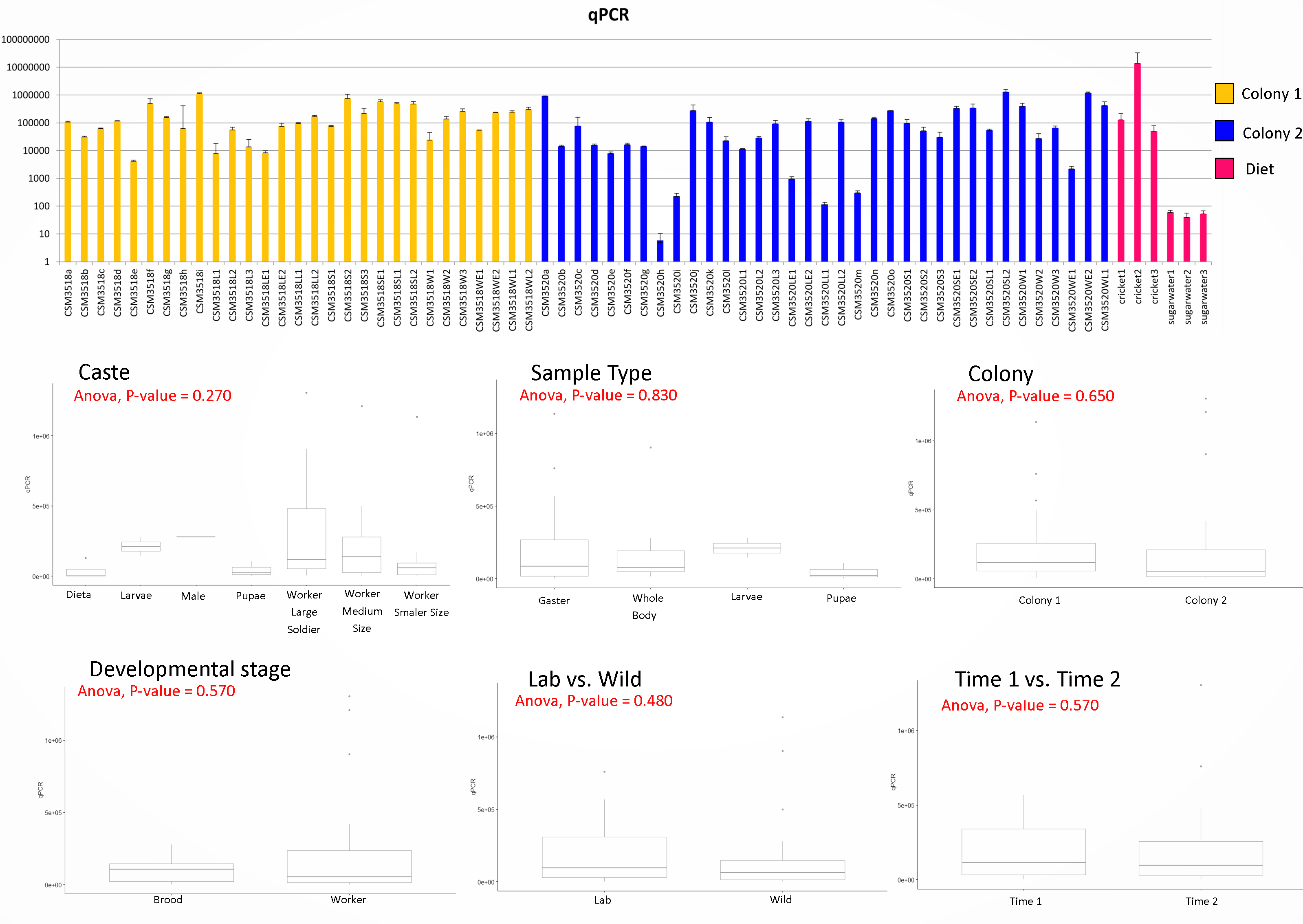

Supplement: Supplementary file 4 — Supplementary Information 4. [file 41598_2020_64393_MOESM4_ESM.tif]

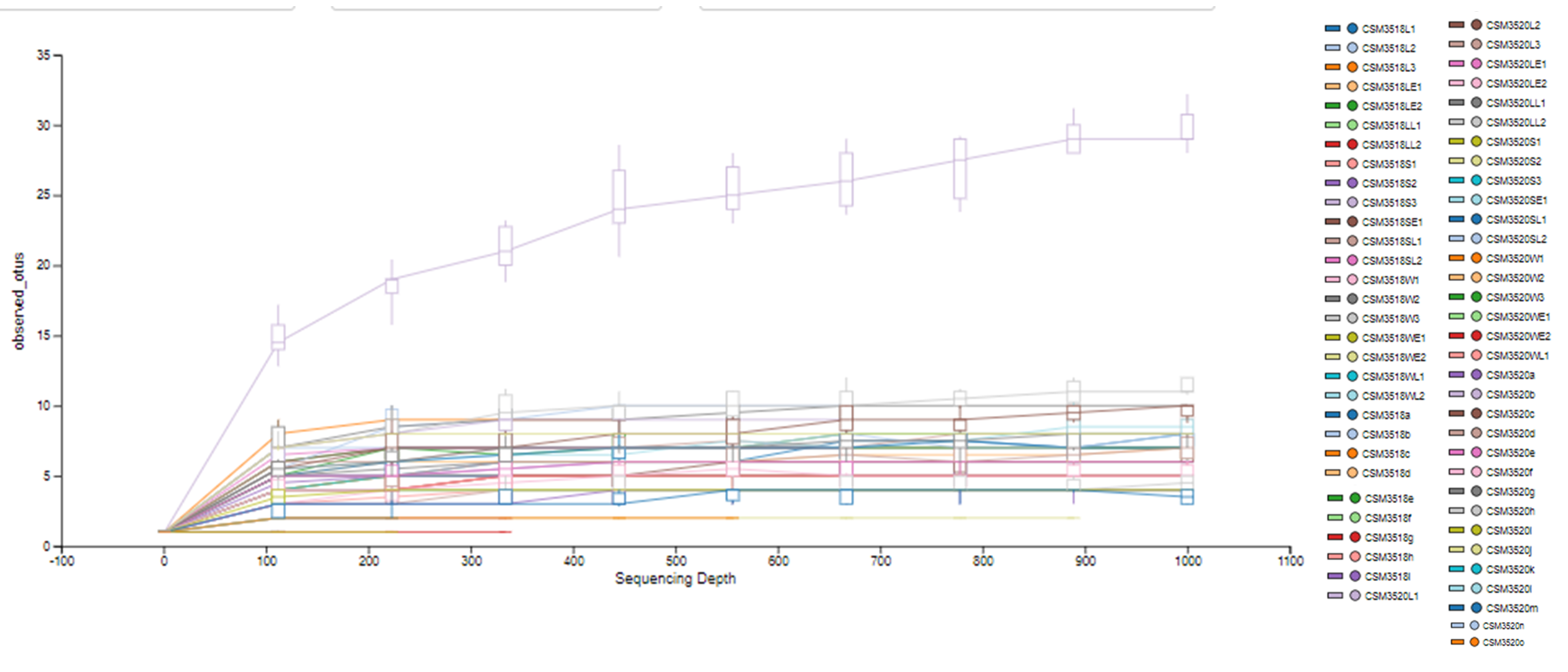

Supplement: Supplementary file 6 — Supplementary Information 6. [file 41598_2020_64393_MOESM6_ESM.tif]

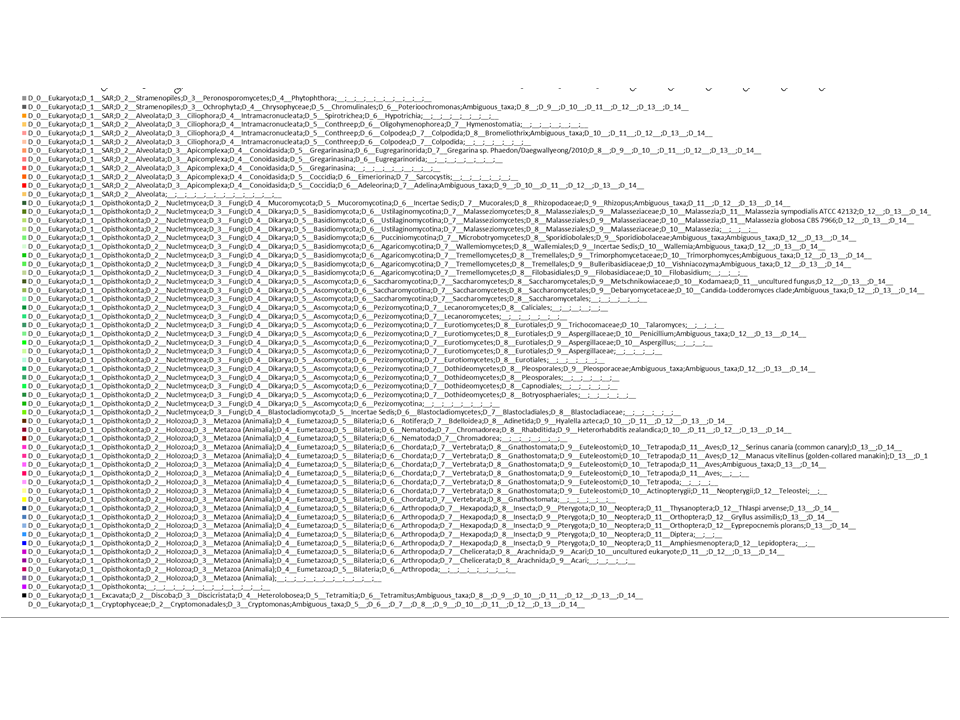

Supplement: Supplementary file 8 — Supplementary Information 8. [file 41598_2020_64393_MOESM8_ESM.tif]
